# Principles of the Guidance of Exploration for Orientation and Specification of Action

**DOI:** 10.3389/fnbeh.2019.00231

**Published:** 2019-10-04

**Authors:** Steven van Andel, Thomas B. McGuckian, Daniel Chalkley, Michael H. Cole, Gert-Jan Pepping

**Affiliations:** School of Behavioural and Health Sciences, Australian Catholic University, Brisbane, QLD, Australia

**Keywords:** affordances, exploration, movement control, general tau theory, action guidance

## Abstract

To control movement of any type, the neural system requires perceptual information to distinguish what actions are possible in any given environment. The behavior aimed at collecting this information, termed “exploration”, is vital for successful movement control. Currently, the main function of exploration is understood in the context of specifying the requirements of the task at hand. To accommodate for agency and action-selection, we propose that this understanding needs to be supplemented with a function of exploration that logically precedes the specification of action requirements with the purpose of discovery of possibilities for action—action orientation. This study aimed to provide evidence for the delineation of exploration for action orientation and exploration for action specification using the principles from “General Tau Theory.” Sixteen male participants volunteered and performed a laboratory-based exploration task. The visual scenes of different task-specific situations were projected on five monitors surrounding the participant. At a predetermined time, the participant received a simulated ball and was asked to respond by indicating where they would next play the ball. Head movements were recorded using inertial sensors as a measure of exploratory activity. It was shown that movement guidance characteristics varied between different head turns as participants moved from exploration for orientation to exploration for action specification. The first head turn in the trial, used for action-orientation, showed later peaks in the velocity profile and harder closure of the movement gap (gap between the start and end of the head-movement) in comparison to the later head turns. However, no differences were found between the first and the final head turn, which we hypothesized are used mainly for action orientation and specification respectively. These results are in support of differences in the function and control of head movement for discovery of opportunities for action (orientation) vs. head movement for specification of task requirements. Both are important for natural movement, yet in experimental settings,orientation is often neglected. Including both orientation and action specification in an experimental design should maximize generalizability of an experiment to natural behavior. Future studies are required to study the neural bases of movement guidance in order to better understand exploration in anticipation of movement.

## Introduction

The understanding of perceptual guidance of movement has developed from research that describes the reciprocal relation between perception and action. Perceptual exploration is a critically important aspect of the ongoing perceptual guidance of movement. It has been described as a function of the neural system charged with collecting information from the organism-environment system (Gibson, 1966, 1979; Reed, 1996). For prospective guidance of action in complex environments, knowledge about *future* opportunities for action is imperative. In terms of affordances—the possibilities for activity in the surrounding environment—this knowledge is expressed relative to an individual as potential future individual-environment relationships. In complex behavioral situations, such relationships give rise to the emergence and dissipation of social synergies that drive individual and social action. Agency and ongoing action can, hence, be understood as emerging from the competition between affordances when multiple actions are afforded simultaneously (Cisek, 2007; Cisek and Kalaska, 2010; Barsingerhorn et al., 2013). In this context, exploratory action, the movements aimed at revealing information about (future) affordances, is of vital importance in driving individual-environment interactions. Previous research on perceptual exploration focusses heavily on the actualization of affordances and hence the specification of action requirements, whilst studies on orientation, i.e., on the behavior aimed at identifying potential future opportunities for action are under-represented. The current study aimed to present evidence supporting the distinction between orienting and specifying exploratory movements in natural behavior.

Perceptual exploration (visual or other) is a basic biological requirement for any species and potentially for life (including that of plants; Carello et al., 2012) in general. It can be seen as a driving force in the context of many human and other animal behavioral traits, such as the evolution of animal locomotion (Gibson, 1958; Warren, 2009), and in humans, of upright locomotion (Rushton et al., 1998; Bruggeman et al., 2007; Rushton, 2008), and the human eye-head-neck musculature (Gibson, 1966; Alexander, 2003; Freedman, 2008). Exploration and the accompanying movement uses energy. Efficient control of exploratory movement by the neural system is thus needed to minimize the metabolic cost of irreversible but idle movements. This efficiency can be achieved through the neural system’s reliance and direct guidance of movement through the perception of specifying optical variables (Lee, 1998). As an example, Lee (1976) showed that human control of braking a car can be regulated by monitoring a single optical variable (named tau) which specifies “time to collision” or “time to closure” of the gap between a moving object and its goal state. Later research showed variations to this strategy to be effective in regulating movement in numerous contexts and different species. For instance, tau was shown to be used by plummeting gannets to control the timing of their wings closing (Lee and Reddish, 1981), to regulate the landing behavior of pigeons (Lee et al., 1993), to guide the movement of single-celled organisms (Delafield-Butt et al., 2012), as well as to regulate the running approach of humans long jumpers (Lee et al., 1982). Whilst these findings may accurately describe the perceptual exploration involved in the regulation of a particular goal-directed movement, it is limited in the sense that it does not account for agency in movement control. That is, it can explain how, for instance, the gannet explores visual information leading to the decision of *when* to close its wings to make a successful dive, but it is limited in explaining *why* the gannet decided on making the diving action in the first place.

In terms of human research, the spatiotemporal organization of exploration has been investigated by Adolph et al. (2000). Based on the spatial and temporal distance from performing an action, different forms of perceptual exploration were proposed to be used. Adolph et al. (2000) showed that toddlers, when confronted with a decision-making task of how to descend a slope, start with visual exploration of the task from a distance. If after this visual exploration they were still unsure of whether they could walk down the slope, they engaged in exploration by touch; e.g., putting a foot on the slope to determine whether the slope was walkable. If the toddlers were still not convinced, they engaged in exploration of alternatives and found different ways of descending the slope (e.g., sitting and sliding down; Adolph, 1995). Adolph et al. (2000) generalized this pattern beyond children on slopes and reasoned that all exploration would follow a similar spatiotemporal organization. The function of exploration described by Adolph et al. (2000) serves the purpose of specifying control requirements of the task at hand, termed here as exploration for *action specification*. What is important to recognize at this stage is that, similar to what has been described for the regulation of goal-directed movement, exploration for action specification does not account for agency in movement control.

Before one can start specifying the demands of the movement to be performed, one must decide what specific action to perform. Hence, exploration of the numerous available possibilities for action is required to select one opportunity for action, or affordance, over all others (Gibson, 1979; Reed, 1996). The current study argues that the understanding of exploration provided through the studies of Adolph (1995) and Adolph et al. (2000) ignores this exploration for different affordances, which we have termed exploration for action-*orientation*. Exploration for action-orientation is required to explore the relevant available options. In the case of the toddlers on slopes, as well as in many other experimental settings, participants are only confronted with one task and no other movement options. Yet, the toddler’s decision to want to walk down the slope already means to ignore an infinite number of other opportunities for action (Withagen et al., 2012) that might also be available. In natural movements, an organism first needs to orient itself toward what actions are available in the environment before deciding what action to perform.

The functional significance of a delineation between exploration for orientation and action specification for species survival can be well illustrated by looking at scenarios in which individuals are completely surrounded by multiple action-opportunities, such as in military, police, surveillance, or sport-situations. In such situations, which are often time-pressured, effective use of both types of exploration is a necessity for successful action. Imagine, for instance, in an invasion sports such as basketball or football, a player in the midfield/court surrounded by both opponents as well as teammates. Before they gain possession of the ball, exploration for orientation is imperative to determine where the ball could come from, where free space exists, where opponent and team players are and where the ball could be passed to once possession is gained. Yet, when the player receives the ball and needs to guide their action to deliver an accurate pass, exploration for action specification is used to collect the required perceptual information to specify for instance how much force is required for the pass. Similarly, in any situation where individuals are fully surrounded by action opportunities, exploration in support of the discovery of response alternatives, i.e., exploration for orientation is imperative and together, both types of exploration are centrally important in determining adequate decision making and skill execution for the player. While the visual information involved in successful movement in sport has been extensively studied in lab-based tasks, this search for information in complex and dynamic, 360-degree environments is not well understood (McGuckian et al., 2018b). Therefore, a better understanding of both types of exploration is required, for instance, in order to design training tools to improve exploration and decision making in practice.

## The Current Study

As a means to assess the characteristics of the control of movement in support of exploration for action-orientation vis-à-vis exploration for action specification, the current study investigated the head movements performed by individuals when they explore their environment in a 360-degree task-environment, such as those encountered in the invasion-sport scenario. Head movements were analyzed in terms of their movement control characteristics, following “General Tau Theory” (Lee, 1998, 2009). This theory has been used to describe regulation of movement in vertebrate as well as invertebrate, multi-cell as well as single-cell biological systems, such as center of pressure movements during human gait initiation (Spencer and van der Meer, 2012; Rasouli et al., 2016; Zhang et al., 2016; van Andel et al., 2019b), club movement in golf putting strokes (Craig et al., 2000a), human neonatal suckling behavior (Craig and Lee, 1999; Craig et al., 2000b), frequency glides in human singing (Schogler et al., 2008), and the guided movement of a free-swimming single-celled organisms (Delafield-Butt et al., 2012). Movement analyses that follow General Tau Theory aim to reveal two important principles of perceptuomotor coupling: (i) the duration of the coupling; and (ii) the (terminal) dynamics of the movement. These two outcomes can be computed if one can clearly define the current state and goal state of a system (with the movement being defined as the closing of the gap between these). It should be noted that in some cases this goal state is clearly defined (e.g., the putter head hitting a golf ball), but that this is not a condition required for the analysis. For instance, gait initiation can be successfully performed with a range safe center of pressure positions, as long as these do not exceed one’s basis of support. The goal state in this situation can be different on a case-by-case basis depending on a person’s intended step length and width, but this can only be inferred *a-posteriori* from an analysis of the center of pressure trajectory. The current study focused on head movements, which have a similar abstract goal state; no specific target is presented and even with a target present, the eyes could compensate if the head is not pointing straight at a visual target (see also Freedman, 2008). This makes a range of head orientations acceptable for any given visual target. However, similar to the case of gait initiation, the goal state of a movement can be inferred from the trajectory of the head movement after it has been performed. In one way, head movements are different from the other movements that have been previously analyzed. That is, when one turns the head to look at something outside their current field of view, the goal of the head movement might not be decided yet, but even in this case, the goal of the movement can be inferred from the movement trajectory.

To provide support for the functional delineation of movement supporting exploration for action-orientation and action specification, the current study aimed to assess the nature of these different types of exploration. It was hypothesized that their different functions would be related to differences in movement patterns and guidance characteristics when animals explore for these different functions (i.e., orientations vis-à-vis specification). When one starts exploring for action-orientation, it is still unknown where the potential end-target is in the surrounding environment. It is therefore likely that exploration for action orientation is characterized by: (i) shorter duration perceptuomotor coupling, only for the latter part of the movement when a visual target is identified; and (ii) a stronger deceleration of the head to provide final head stability for a successful eye fixation. Conversely, after orientation, once the animal is aware of the location of a visual end-target and is in need of exploration for action-specification, we hypothesized that the movement will be characterized by: (i) longer duration perceptuomotor coupling; and (ii) as errors in action-specification might influence the performance of the following movement and performers will be seeking to avoid overshooting, we expect to find earlier velocity peaks and smoother decelerations/lower terminal velocities (less abrupt ending) in exploration for action-specification.

## Materials and Methods

### Participants

Sixteen participants volunteered in the study (mean age: 17.25; SD: 0.77). All participants were free of injury, had at least 9 years of playing experience in playing youth football and were active in the youth teams of an Australian semi-professional football club. The research was approved by the Australian Catholic University’s Human Research Ethics committee and all participants provided written informed consent. When participants were under the age of 18, written informed consent as well as written informed assent was obtained from the parents or guardians of the participants.

### Design

Participants were presented with a laboratory-based visual exploration task based on an association-football (soccer) in-game passing situation. Five computer monitors situated on top of five tables that were arranged surrounding the participant, these were used to show a series of in-game situations. The main monitor (a 15-inch laptop, Apple, Inc., Cupertino, CA, USA) was placed in front of the participant. The other four screens (22-inch Dell 2209WA, Round Rock, TX, USA) were positioned on the surrounding tables (at 100 degrees and 150 degrees to the left and right of the participant), and were set to portrait position, yielding the same visual angle of the screen to the visual angle of an 180 cm tall player standing at an 11.5 m distance. Four cones were positioned on the ground in front of these screens. A kick of one the cones represented one of four response options (see below).

A custom made PsychoPy (Peirce, 2007, 2009) script was used to present a virtual passing scenario to the participant. The participant started the trial by pressing the spacebar on the keyboard near the main screen and after a random time interval (between 1 and 4 s) all five screens would start playing a different video as part of the passing scenario. The four surrounding screens presented one of four situations; open space, a free teammate, an opponent or a marked teammate. The main screen positioned in front of the participant showed a video of a teammate passing them the ball (left or right footed pass with a delay of 1, 2 or 3 s). The participant was instructed to explore the response options, with no instructions given as to how they should explore. When the ball was “passed” (left the bottom edge of the main screen) and the participant was “in possession,” they were instructed to kick the cone in front of the screen to which they would choose to play the ball. The participants first completed five practice trials, after which they completed four blocks of 24 trials, with 5 min of rest in between. The protocol took approximately 50 min.

Head movements were monitored using a 9-degrees-of-freedom Inertial Measurement Unit (IMU) sensor (SABELSense, Nathan, QLD, Australia) sampling at 250 Hz that was fitted over the occipital protuberance of the skull using an elastic headband. A custom program, written in MATLAB (R2018a, MathWorks Inc., Natick, MA, USA) was used to process and analyze IMU recordings (Chalkley et al., 2018). This program uses an attitude and heading reference system (AHRS) algorithm to obtain head orientation and angular velocity (Madgwick et al., 2011) from the raw IMU channels. A head turn was defined as a horizontal movement of the head in space, with an angular velocity greater than 125 degrees/second (McGuckian et al., 2018a, 2019).

**Figure 1:**
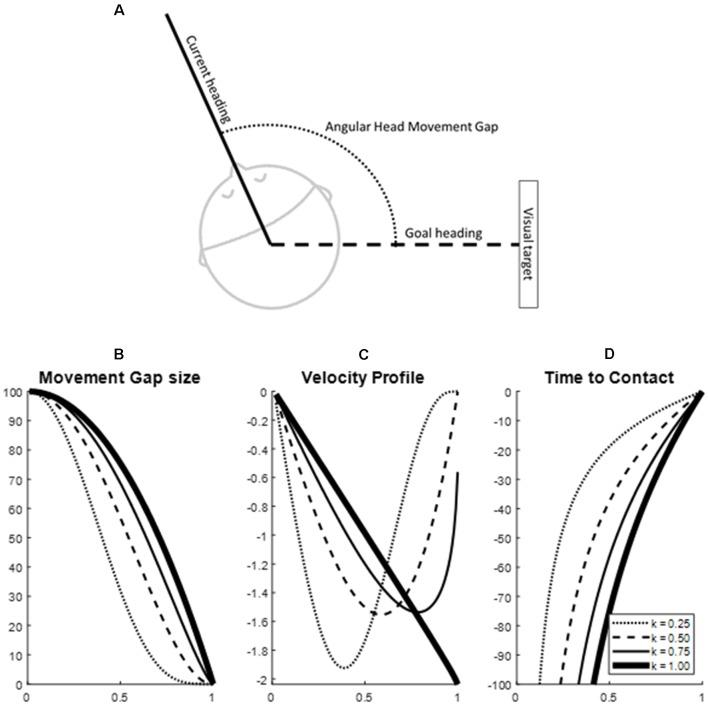
Example of movement guidance characteristics in a head movement of 100 degrees over 1 s (simulated data). Panel **(A)** depicts the identification of a movement gap in head movement. Panels **(B–D)** depict the tau-coupling with four different coupling constants (*K*-values). Specifically, panel **(B)** depicts the closure of the movement gap in time, panel **(C)** depicts the velocity profile and panel **(D)** the time to contact. The solid line in panels **(B–D)** indicates a one-on-one coupling with the tau guide function (Coupling constant *K* = 1). Note how lower *K*-values lead to earlier peaks in the velocity profile and softer closures of the movement gap.

The head movement was described in terms of the time to closure (tau) characteristics of the head movement gap. In the case of the current study, the head movement gap was defined as the difference between the current positioning of the head (Figure 1A) and the goal position, with the time-to-closure of the gap defined as “*tau_movement_*.” Using general tau theory, differences in characteristics of the velocity profile of the gap closure are expressed in one value, the coupling factor with a reference movement or “*tau*_guide_” (Lee, 1998). Movement can be effectively guided by keeping *tau*_movement_ proportional to *tau*_guide_ following the formula *tau*_movement_ = K*tau_guide_. In which the *tau*_guide_ represents the time to closure of a canonical energy gap with a constant acceleration and is coupled to *tau*_movement_ by coupling constant *K*. The coupling constant *K* between the *tau*_movement_ and *tau*_guide_ summarizes the velocity profile characteristics during the gap-closure (Figures 1B–D). A *K* of less than 0.5 indicates an early velocity peak and longer deceleration of the movement, leading to soft closure of the gap with no residual velocity; a *K* between 0.5 and 1 indicates a later velocity peak and closure of the gap that includes contact that is increasingly harder with a higher *K*; and finally, a *K* of more than 1 indicates a constant or increasing acceleration in the movement and thus an overshooting of the movement (Lee, 1998, 2009). An analysis of the tau-guidance characteristics leads to two outcome variables: (i) the coupling value *K* which summarizes the velocity profile and the type of contact (i.e., zero velocity contact, non-zero velocity contact, or hard contact) of the movement gap closure, and (ii) the proportion of a movement that keeps the tau of the movement proportional to the *tau*_guide_, indicating to what degree the control of the movement follows the principles of tau-guidance.

### Dependent Variables

Each head movement was analyzed in terms of its kinematics and tau-guidance characteristics. In order to analyze the kinematics of the head movements, *the total angle, movement time and peak velocity* were computed. Furthermore, the tau-guidance characteristics were computed as follows. The motion gap at any moment (t) in the head turn was defined as the gap between any angular position during the movement and the final angular position in the head turn. Using the angular velocity profile of the closing of this gap, the time to closure given the current velocity (*τ*_HM_) was computed. The tau-guide (*τ*_G_) describes the closure of a gap with a constant acceleration and can be mathematically represented as the function *τ*_G_ = 0.5 (*t* − *T*_G_^2^/*t*) (Lee, 1998, 2009). In this function, *T*_G_ is the total time taken to close the motion gap and *t* represents the timing within the movement which runs from 0 to *T*_G_. Considering the possibility that not the entire movement follows a guidance strategy, but potentially only the final part does, a recursive linear regression was used to establish the coupling between *τ*_G_ and *τ*_HM_. If the *R*^2^ calculated between *τ*_G_ and *τ*_HM_ was smaller than 0.95, the data point furthest from the gap-closure was removed from the analysis and the regression was recalculated. This process was repeated until an *R*^2^ of at least 0.95 was reached. Resulting from the recursive regression, the final *coupling constant* K and the percentage of the movement that yielded an *R*^2^ of higher than 0.95 (*percentage tau-coupled*) were used in the statistical analysis as dependent variables.

### Analysis

All head movements were detected throughout the trials using the IMU sensors. Analysis focused firstly on the head movements (HM) that occurred right after the presentation of the visual scene, which were coded in order of appearance (HM1, HM2… HM*n*; for the first, second and *n*’th head turn after the trial start). After the ball had left the bottom edge of the main screen and the participant was “in possession,” the player responded by kicking the cone in front of the screen to which they would choose to play the ball. The head movement just before the initiation of the kick-response was established as the final head-movement (HMf). Using this design, HM1 (and to a lesser extent HM2, etc.) is hypothesized to show characteristics of the orientation function of exploration, as this is the first instance when participants start to familiarize themselves with the visual scene. As such, orientation would become less and less of a factor as *n* increases and therefore HMf is hypothesized to show the least characteristics of orientation. In contrast, exploration for specification becomes more important as the trial progresses and was hypothesized to be mostly observable in HMf as this is the final head movement before a response is made.

The aims for the statistical analysis were two-fold. First, it was aimed to contrast the characteristics between exploration for orientation and exploration for specification. To this end, a paired samples *t*-tests were performed on the set of dependent variables (Head Movement Angle, Movement Time, Peak Velocity, Percentage tau-coupled and Coupling Constant K). Furthermore, in order to assess how exploration changes as the focus shifts from orientation to specification, Linear Mixed Effects (LME) modeling was used. The analysis focused on the relationship between the dependent variables and the sequence of head turn occurrence. In the analysis, modeling of the dependent variable was performed by allowing different intercepts per head movement number. To this end, a LME model was designed for each dependent variable, in which the HMn was included as a random factor, with HM1 being the first head movement in a trial and HMn being the *n*’th movement (note that only head movements before ball reception are included). Five different models were assessed for the different dependent variables: Head Movement Angle, Movement Time, Peak Velocity, Percentage tau-coupled and Coupling Constant K.

## Results

**Figure 2:**
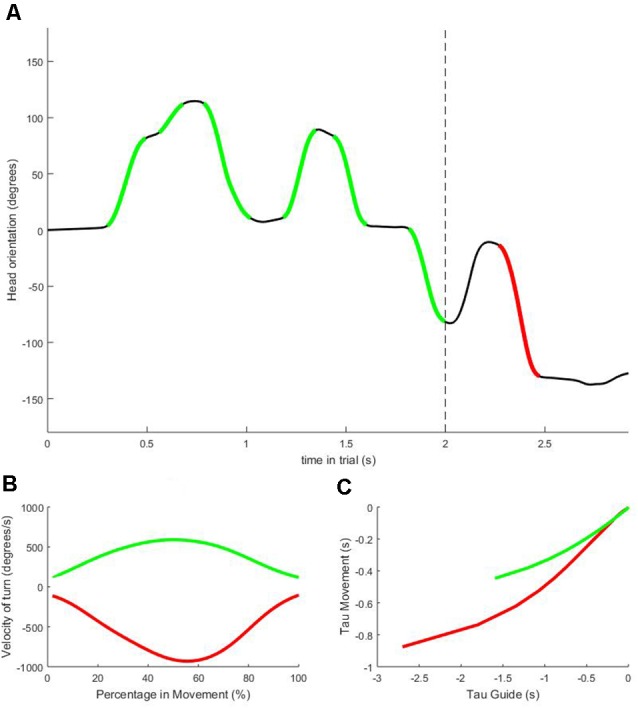
Sample data from one representative trial. Panel **(A)** shows head orientation with 0 degrees indicating the head positioned straight towards the central screen. The dashed vertical line indicates the moment the virtual ball is played, and the player gains possession. All head movements detected before possession (highlighted in green) are numbered in order of occurrence (HM1, HM2… HMn). The final head movement before the response is given (highlighted in red) is labeled HMf. Panel **(B)** shows the velocity profiles of the very first (HM1; green) and last (HMf; red) head movement depicted in panel **(A)**. Panel **(C)** shows the relation between the Tau-guide and the Movement Tau for HM1 (green) and HMf (red). The steeper line for HMf indicates a higher value of coupling constant *K* for this head movement compared to the depicted HM1.

On average, participants performed 3.51 (SD 1.90) head movements before ball-possession per trial. The computations required to extract the dependent and independent variables from the IMU data for each trial are illustrated in Figure 2. Paired samples *t*-tests were used to investigate the different characteristics between HM1 and HMf, the results are shown in Table 1. No significant differences were observed between HM1 and HMf for any of the dependent variables (all *p-values* > 0.05).

**Table 1:** Results of paired *T*-test between the first (HM1) and last (HMf) head movement (*df* = 15).

	Mean value			
	HM1	HMf	*t*-stat	*p*-value
Angle (degrees)	85.73	85.93	−0.036	0.972
Movement time (seconds)	0.24	0.23	1.224	0.240
Peak velocity (degrees/second)	200.40	241.33	−0.925	0.370
Coupling constant *K*	84.04	84.23	−0.068	0.947
Percentage tau-coupled	0.43	0.40	0.872	0.397

The LME modeling analysis was used to get better insights of how participants progressed through the orientation phase, as their aim shifts from orientation to specification. In order to interpret the results for this analysis, it should be noted that the LME only identifies what conditions (Head Movement numbers) can be modeled with a coefficient significantly different from the mean of all head movements. As such, the LME provides information about what condition is different from the mean but does not contrasts any two specific conditions.

**Figure 3:**
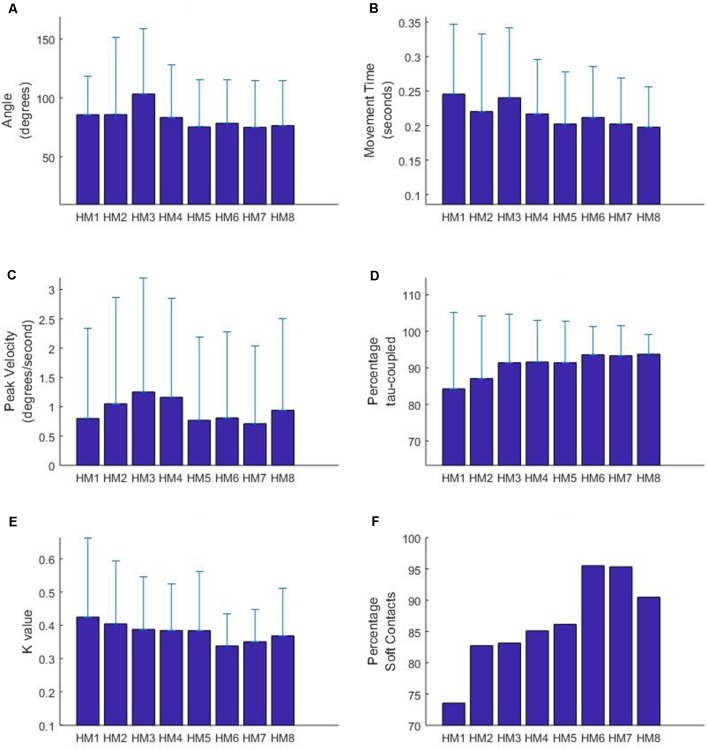
Characteristics of head movements occurring before the participant gained possession of the virtual ball. HM9 to HM11 are not depicted due to a limited sample size (less than 10 observations overall participants). Figure displays means and standard deviations for movement angle **(A)**, movement time **(B)**, peak velocity **(C)**, percentage tau-coupled **(D)** and coupling constant *K* **(E)**. Panel **(F)** displays the percentage of head movements with a “soft” gap closure, indicated by a *K*-value of less than 0.5.

The results for the LME analysis are summarized in Figure 3 and the statistics are reported in Table 2. In terms of the total angle of the movement, it appears that HM3 (larger) and HM5 (smaller) are significantly different from the mean head movement angle. In terms of movement time, differences are found for HM1, HM3 (longer) and HM5 (shorter) and in terms of peak velocity HM3 and 4 are found to have a higher velocity. Some of these results are obviously interrelated; for instance, HM5 has both a small angular size as well as a short movement time. Furthermore, some of these results can be related to the experimental design, with two targets on either side. The finding that HM3 is significantly different in terms of angle, movement time and velocity can be related to exploring the two targets on one side with the first two movement and then turning the head to the other side using HM3.

**Table 2:** Results from the linear mixed effects modeling analysis.

	Coefficients in LME analysis										
	HM1	HM2	HM3	HM4	HM5	HM6	HM7	HM8	HM9	HM10	HM11
Number of observations in analysis	1,392	1,170	801	604	397	223	107	42	7	1	1
Angle	1.478	1.614	**18.326**	−0.786	**−8.132**	−5.009	−6.941	−4.268	2.434	0.285	0.998
Movement time	**0.026**	0.001	**0.020**	−0.002	**−0.015**	−0.006	−0.012	−0.011	0.000	0.000	0.001
Peak velocity	−0.147	0.086	**0.263**	**0.177**	−0.151	−0.103	−0.130	−0.005	0.003	−0.015	0.023
Percentage tau-coupled	**−6.299**	**−3.548**	0.679	0.851	0.684	2.577	2.068	1.821	0.827	0.182	0.158
Coupling constant *K*	**0.040**	0.020	0.004	0.001	0.000	**−0.035**	−0.021	−0.006	−0.003	0.001	0.000

*Note. Coefficient significantly different from 0 (at alpha = 0.05) are presented boldfaced and red*.

In terms of the tau-guidance characteristics, there seems to be a trend for a lower percentage coupling and higher *K*-values as orientation commences. That is, HM1 and HM2 were found to be significantly different in terms of the percentage tau-coupling (with lower percentages) and HM1 (higher) and HM6 (lower) were different in terms of *K*-values. Furthermore, Figure 3E illustrates the increase in the number of head movements with “soft contact” (i.e., a gradual deceleration in the final phase of movement, characterized by a *K* value smaller than 0.5), as participants progressed through the orientation part of the experiment.

## Discussion

Visual exploration is a basic biological function, providing the neural system with the inputs required for the successful guidance of action. It was proposed that human exploratory head movements, executed to achieve different goals—i.e., to discover opportunities for action (orientation) vs. to specify end-task requirements (specification), are fundamentally different in terms of their movement control characteristics. To this end, an experiment was designed that first afforded the participant to explore for orientation, followed by a need for action guidance. Head movements that were thought to be associated with exploration for orientation and head movements associated with exploration for action specification were contrasted in terms of their movement guidance characteristics. It was hypothesized that exploration for orientation would be characterized by a low percentage of the movement adhering to the tau-guidance principles and a late peak in the velocity profile followed by strong decelerations (i.e., hard gap closures) and vice versa for exploration for action specification.

It should be considered that any particular head movement might not have the sole purpose of orientation or specification, but that they likely serve a mixed purpose. As such, in our experiment, we reason that the first head movement (HM1) was likely most associated with orientation and that the dominance of orientation vs. specification would slowly change in the following head movements. In contrast, the final head movement (HMf) can be reasoned to be mostly associated with specification, but it cannot be ruled out that it would still serve the aim of orientation as well. Considering this, the results of the LME analysis were in accordance with the hypothesis; showing a low percentage tau-coupling and higher *K*-values in the first head movement, and these effects lessening in the following movements. However, these results were not confirmed in the analysis that directly contrasted the first and final head movement. In fact, no differences were found in movement angle, time, peak velocity or tau-guidance characteristics between the first and last head movement in the trial.

It is interesting to find confirmation of the hypothesis within head movements performed before the participant was “in possession” of the ball, but not when contrasting the two head movements that theoretically should have yielded the strongest effect (i.e., the first and last in the trial). This absence of differences between movement characteristics could potentially be explained by the nature of the trial. Potentially, the current experimental design did not afford exploration for action specification as much as was intended with the protocol. Indeed, when looking at the type of skills often used to study this type of exploration, these skills often require a fixation on a distant target (known as quiet eye; Vickers, 2007, 2016). In the protocol used here, a response action was required of the participant in the form of kicking a cone to signal one of four passing directions. As such, the participant only needed to specify the position of the cone. Information normally obtained using “quiet eye” about for instance distance to and direction of a target did not need to be visually specified. It is likely that in reducing the complexity of the natural environment to adjust the task to the laboratory environment, we reduced the invitation for real action specification in the final head turn.

Further study is required to fully contrast exploration for orientation and specification in a more representative task; ideally in the actual performance setting. Historically, this has been difficult, as technology required for these analysis (i.e., motion capture technology to analyze head movement or eye-tracking for visual exploration) has always been confined to the laboratory environment (McGuckian et al., 2018b). With the use of Inertial Measurement Units (IMUs), the current study administered novel technology which allows visual exploration to be recorded in any environment. It is a limitation of this technology that it only records head movements, without monitoring activity of the eyes. However, visual exploration does not stop at the eyes; the head, torso and the entire body can be involved in exploration (Gibson, 1966, 1979; Soska and Adolph, 2014; Warman et al., 2019). In fact, research has shown that gaze shifts greater than 25 degrees are usually associated with a head turn (Freedman and Sparks, 1997), illustrating the association between head and eye movement (Freedman, 2008). Animal research has provided evidence for a neuroanatomical link between eye and head movements (Hadjidimitrakis et al., 2007). It can, therefore, be considered that head movements can be used as a valid proxy for visual exploration in general.

In the practice environment, it could be hypothesized that the effects of action orientation and specification are related to the changing demands of the environment. For instance, in football, when the dynamics of play rapidly change (imagine a midfielder in defense, gaining possession of the ball at which point all his teammates start attacking), there is a strong need for orientation. Yet when play is less dynamic (imagine a team attacking for minutes on end, only playing broad passes at half field), orientation would be less necessary. We would reason that perceiving these task demands and producing an adequate response in terms of exploration is indicative of a good situational awareness (Smith and Hancock, 1995; Stanton, 2010). The current study could form the foundation of new assessment tools to monitor football player’s situational awareness and potentially to inform and evaluate training interventions aimed at increasing situational awareness.

The different nature of the orientation and the action specifying head turn refers to the concept of nested affordances (Reed, 1996; Stoffregen, 2003; Smith and Pepping, 2010; Wagman and Morgan, 2010). This concept indicates that actions are embedded in one another. An action specification head turn is only possible when a person is already familiar with the general locations of targets and one can use a more controlled movement to fixate gaze on the target; action specifying exploration is embedded in orientation exploration. With this background, it is noteworthy that research on visual guidance of action predominantly focusses on exploration for action specification, whilst this is nested within exploration for orientation. Current understanding of exploration (Adolph et al., 2000) encompasses a nested structure, led by the spatiotemporal organization in relation to the task. The current study reasoned that this understanding can be broadened to account for agency. In the example of Adolph et al. (2000), a toddler is only able to judge the descend-ability of a slope after it has oriented and identified the presence of a slope. Our results show that even within this phase of exploration, movements of a different nature occur. These different aims of exploration should, therefore, be represented as a separate phase in a model aimed for understanding exploration in the service of prospective control. It is a general recommendation for future studies to account for action-orientation in experimental design, in order to generate behavior that is more representative of natural behavior.

The insights gained from the current study can be generalized to visual exploration in a broader context. For instance, this understanding of the difference between exploration for action-orientation and exploration for action-specification can be used to explain some seemingly contradictory findings in research on the visual guidance of locomotion. A previous study in this field has shown that in successfully regulating foot placement in human locomotion, one most relies on information from at least two steps ahead of the current position, but not closer (Matthis and Fajen, 2011). In contrast, another study (Young and Hollands, 2010) showed that if older adults were required to fixate on foot placement targets until contact was made with their foot, accuracy in foot placements increased. The first type, that seems to end on two steps distance, would be related to orientation in the environment and can be used to identify possible targets as well as obstacles for locomotion (Gibson, 1958). The latter type of exploration, holding visual contact with a target as long as possible, might be related to action specification; the fixation on obstacles in the planning of movement (Rodrigues and Navarro, 2016; Vickers, 2016). Interestingly, a recent study by Ellmers et al. (2019) showed that older adults at a high risk of falling focus their visual exploration only on the immediate path in front of them. The authors reasoned that this reduced “planning.” In our terminology, the results can be interpreted to refer to reduced exploration for orientation, to impact safety during walking. As such, monitoring these different types of exploration in older adults with an elevated falls risk might lead to better understand falling and falls prevention.

Though “General Tau Theory” has been around since the late nineties (Lee, 1998), the use of a tau-guidance analysis in the study of human head movement is novel. This novel approach leads to a better understanding of perceptual exploration in the service of agency and prospective control. As both orientation and action specification are required for successful control of movement, the enhancement of individual exploration strategies could have important implications for the practice environment. For instance, this could lead to interventions improving decision making on the sports field (McGuckian et al., 2019) or the perceptual guidance of gait in older adults (van Andel et al., 2018, 2019a).

In summary, exploration is the neural system’s means to gather the information required for the guidance of action. It was proposed that the current understanding of exploration in service of prospective control was insufficiently capable of accounting for agency for organisms in environments filled with possibilities for action. It was hypothesized that exploration for action-orientation should be considered as a precursor for the more commonly studied exploration for action-specification and that, in the process of exploration, one would slowly move from orientation to specification. An analysis of the movement guidance characteristics of head turns used for orientation and action-specification was presented. Results showed that the first head movement when participants start exploring was characterized by a peak velocity late in the movement, followed by a strong deceleration. Head movements that followed after this first would be more and more associated with specification. These head movements were characterized by earlier velocity peaks and a more controlled movement. These results add new understanding to how the neural system assembles the perceptual information that is used for agency and the successful prospective guidance of action.

## Data Availability Statement

The datasets generated for this study are available on request to the corresponding author.

## Ethics Statement

The research was approved by the Australian Catholic University’s Human Research Ethics committee and all participants (and where appropriate their guardians) provided written consent.

## Author Contributions

TM, MC and G-JP were responsible in study design and planning. TM and DC were responsible for data collection. G-JP, SA and TM conceived the original idea for the concept and data analysis presented in the manuscript. SA was responsible for data analysis and writing the first draft of the manuscript. All authors were involved in proofing the manuscript. SA and G-JP were involved in finalizing the manuscript for submission.

## Conflict of Interest

The authors declare that the research was conducted in the absence of any commercial or financial relationships that could be construed as a potential conflict of interest.
